# Sumoylation of CCAAT/enhancer-binding protein α is implicated in hematopoietic stem/progenitor cell development through regulating *runx1* in zebrafish

**DOI:** 10.1038/srep09011

**Published:** 2015-03-11

**Authors:** Hao Yuan, Tao Zhang, Xiaohui Liu, Min Deng, Wenqing Zhang, Zilong Wen, Saijuan Chen, Zhu Chen, Hugues de The, Jun Zhou, Jun Zhu

**Affiliations:** 1 CNRS-LIA124, Sino-French Research Center for Life Sciences and Genomics, State Key Laboratory of Medical Genomics, Rui-Jin Hospital, Shanghai Jiao Tong University School of Medicine, Shanghai, China; 2 Laboratory of Development and Diseases, Institute of Health Sciences, Shanghai Institutes for Biological Sciences and Graduate School, Chinese Academy of Sciences, Shanghai, China; 3 Laboratory of Zebrafish Modeling and Drug Screening for Human Diseases of Guangdong Higher Education Institutes, Department of Cell Biology, Southern Medical University, Guangzhou, China; 4 State Key Laboratory of Molecular Neuroscience, Division of Life Science, Hong Kong University of Science and Technology, Hong Kong, China; 5 Université de Paris 7/INSERM/CNRS UMR 944/7212, Equipe Labellisée No. 11 Ligue Nationale Contre le Cancer, Hôpital St. Louis, Paris, France

## Abstract

The small ubiquitin-related modifier (SUMO) participates in various cellular processes, including maintenance of genome integrity, nuclear transport, transcription and signal transduction. However, the biological function of sumoylation in hematopoiesis has not been fully explored. We show here that definitive hematopoietic stem/progenitor cells (HSPCs) are depleted in SUMO-deficient zebrafish embryos. Impairment of sumoylation attenuates HSPC generation and proliferation. The hyposumoylation triggered HSPC defects are CCAAT/enhancer-binding protein α (C/ebpα) dependent. Critically, a SUMO-C/ebpα fusion rescues the defective hematopoiesis in SUMO-deficient embryos, at least in part through restored *runx1* expression. While C/ebpα-dependent transcription is involved in myeloid differentiation, our studies here reveal that C/ebpα sumoylation is essential for HSPC development during definitive hematopoiesis.

In vertebrates, hematopoiesis is a dynamic process involving primitive and definitive waves. Primitive hematopoiesis is transitory and mainly produces erythrocytes and macrophages. Definitive hematopoiesis generates self-renewing pluripotent HSPCs capable of giving rise to all blood cell lineages^1^. Over the last decade, the zebrafish (*Danio rerio*) has emerged as an excellent model organism in hematopoiesis research^2^,^3^,^4^. In zebrafish, the primitive wave of hematopoiesis gives rise to myeloid cells in the anterior lateral mesoderm (ALM) and to mostly erythrocytes and some myeloid cells in the posterior lateral mesoderm (PLM), which later becomes the intermediate cell mass (ICM) at 18 hours post fertilization (hpf). The definitive wave of hematopoiesis that contains HSPCs originates in the aorta-gonad-mesonephros (AGM) region at 30 hpf. Subsequently, the HSPCs migrate to the caudal hematopoietic tissue (CHT) in the posterior region of the tail at 48 hpf, and finally colonize the kidney where adult hematopoiesis occurs at 4 days post fertilization (dpf)^5^. Hematopoiesis is evolutionarily conserved from zebrafish to mammals. The AGM, the CHT and the kidney of zebrafish are functional analogs to the AGM, the fetal liver and the bone marrow of mammals, respectively^6^.

The SUMO proteins belong to the growing family of ubiquitin-like proteins (UBLs) involved in posttranslational modification. Sumoylation is carried out by a multistep enzymatic cascade reaction consisting of a SUMO-activating enzyme (E1), a heterodimer of Sae1 and Sae2, a unique SUMO-conjugating enzyme (E2), Ubc9, and SUMO ligases (E3), which facilitate attachment of SUMO to the substrates^7^. In vertebrates, at least three SUMO paralogues have been identified, designated SUMO1-3. Sumoylation regulates a wide variety of cellular processes such as transcription, DNA repair, trafficking and signal transduction^8^. Our previous work showed that sumoylation played an important role in the myelo-erythroid progenitor cell (MPC) fate decision during primitive hematopoiesis of zebrafish^9^. However, the functional role of sumoylation during definitive hematopoiesis is still unknown.

Here we show that loss of SUMO conjugation results in decreased numbers of HSPC. The impairment of HSPC in SUMO-deficient embryos is due to reduced HSPC generation and proliferation. Mechanistically, hyposumoylation-triggered HSPC depletion is C/ebpα dependent and a SUMO-C/ebpα fusion protein can restore defective definitive hematopoiesis. Thus, the fine-tuning of C/ebpα sumoylation is critical for definitive HSPCs homeostasis.

## Results

### Loss of SUMO leads to definitive hematopoiesis defects

**Figure 1:**
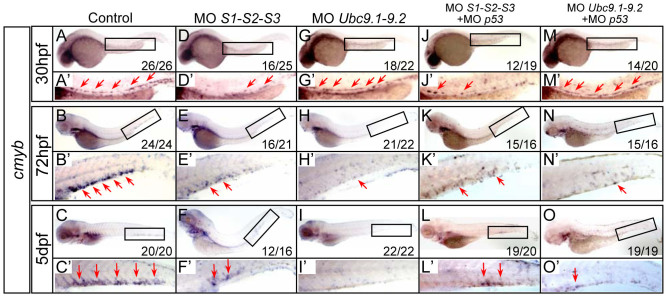
HSPCs are depleted in SUMO-deficient embryos. (A–O) WISH assay of *cmyb* at 30 hpf (A, D, G, J, M), 72 hpf (B, E, H, K, N) and 5 dpf (C, F, I, L, O). Boxed regions indicate the AGM or CHT, respectively. (A′–O′) Magnified images of corresponding boxed regions from A to O, respectively. Red arrows identify *cmyb*-positive cells in the AGM or CHT, respectively. MO, morpholino oligonucleotides.

As our previous studies demonstrated that lack of sumoylation biased primitive hematopoiesis^9^, here we questioned its role in definitive hematopoiesis. To evaluate the effects of sumoylation on definitive hematopoietic development, the temporal and spatial expression patterns of a panel of HSPC and lineage-specific markers were examined by whole-mount mRNA *in situ* hybridization (WISH) after antisense MO-mediated knock-down. The MOs efficacy has been successfully validated previously^10^,^11^. Compared with control embryos (Fig. 1 A–C), the expression of *cmyb*, a marker of HSPCs, was strikingly diminished in the AGM from 30 hpf onward in SUMO-deficient embryos (Fig. 1D), and almost no *cmyb* signals were detected in the CHT at 5 dpf (Fig. 1F), consistent with the phenotypes observed in the *sae1* mutant, a SUMO E1 mutant line^12^. Similarly, the expression of another HSPCs marker, *runx1*, was also markedly decreased in the AGM in SUMOs morphants (Supplementary Fig. 1A). Two *Ubc9* genes (*Ubc9.1* and *Ubc9.2*), encoding a unique SUMO-conjugating enzyme of sumoylation pathway, have been identified in zebrafish^11^,^13^. As expected, knockdown of *Ubc9* also led to depleted HSPCs in the CHT, as indicated by reduced *cmyb* expression at 72 hpf and 5 dpf (Fig. 1 H–I). The *cmyb* expression level was relatively unchanged in *Ubc9* morphants up to 30 hpf (Fig. 1G), possibly due to maternal Ubc9 protein^11^. Consistent with WISH data, loss of SUMOs or Ubc9 resulted in considerably reduced *cmyb*-EGFP positive cells in the CHT of a Tg (*cmyb*-EGFP) transgenic line (Supplementary Fig. 1 B–C). To rule out the MOs off-target effects mediated through p53 activation^14^, SUMOs or *Ubc9* MO was injected with *p53* MO or into *p53* null mutants, respectively. Similar phenotypes were observed in the co-injected embryos or *p53* null mutants (Fig. 1 J–O and Supplementary Fig. 1D). Collectively, these data indicate that impairment of sumoylation leads to depletion of HSPCs during definitive hematopoiesis.

**Figure 2:**
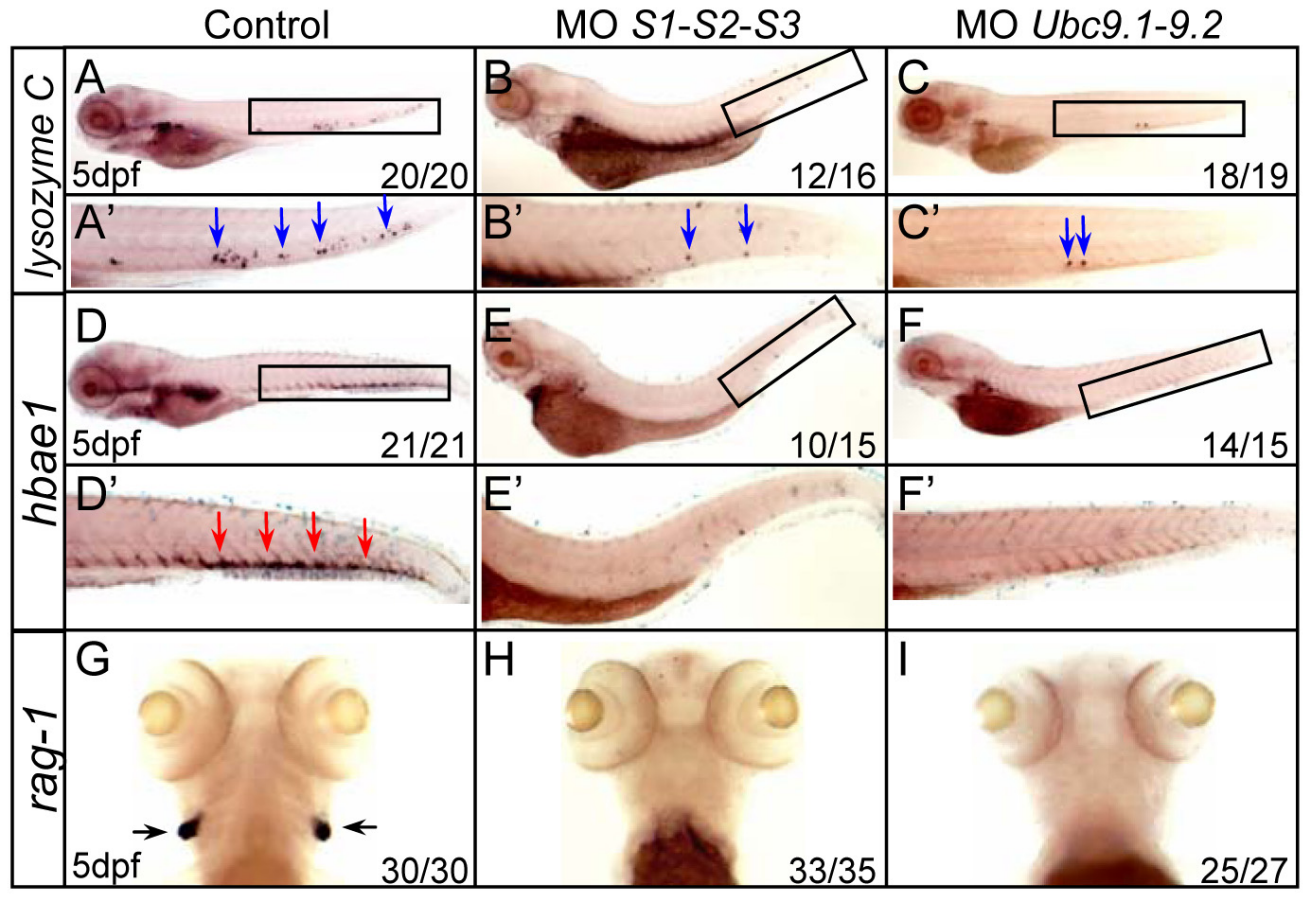
Multiple blood cell lineages are impaired in SUMO-deficient embryos. (A–I) WISH assays of *lysozyme C* (A–C), *hbae1* (D–F) and *rag1* (G–I) at 5 dpf. Boxed regions indicate the CHT (A–F). (A′–F′) Magnified images of corresponding boxed regions from A to F, respectively. Blue arrows identify *lysozyme C*-positive cells in the CHT (A′–C′). Red arrows identify *hbae1*-positive cells in the CHT (D′–F′). Black arrows identify *rag1*-positive cells in the thymus (G–I).

To further confirm that HSPCs were depleted in SUMO-deficient embryos, lineage-specific markers related to definitive hematopoiesis were examined. Expression of the myeloid lineage marker *lysozyme C* and erythroid lineage marker *hbae1* were drastically decreased in both SUMOs and *Ubc9* morphants (Fig. 2 A–F). Expression of the lymphoid lineage marker *rag1* was absent in the developing thymus compared with control siblings (Fig. 2 G–I). Thus, these results demonstrate that the three major definitive hematopoitic lineages are all compromised in SUMO-deficient embryos.

### The defects of definitive hematopoiesis are due to reduced HSPC generation and proliferation

**Figure 3:**
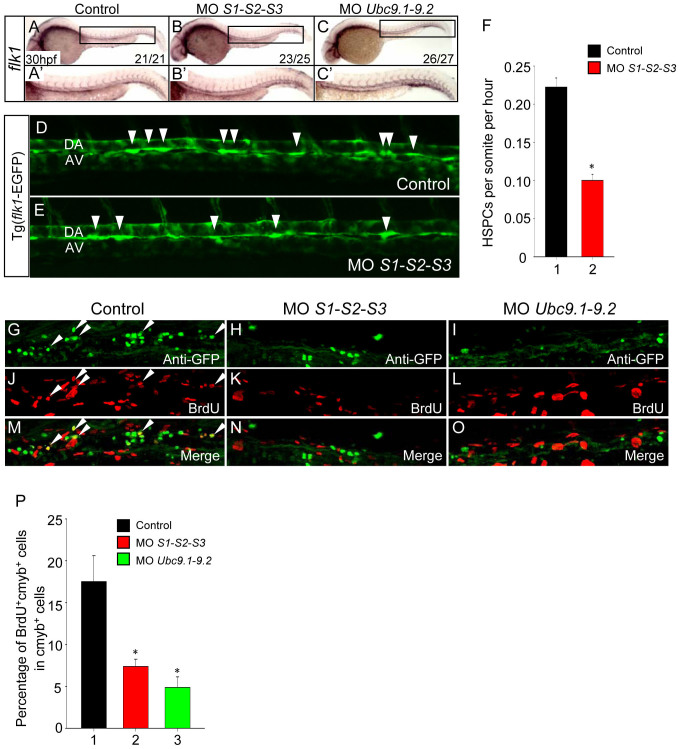
Loss of SUMO reduces HSPC generation and proliferation. (A–C) The vasculature is intact in SUMOs or *Ubc9* morphants. WISH assay of *flk1* at 30 hpf. (A′–C′) Magnified images of corresponding boxed regions from A to C, respectively. (D–E) The frequency of HSPC generation was reduced in SUMO-deficient embryos. Time-lapse confocal imaging analyses of HSPC generation from Tg (*flk1*-EGFP) line from 30 to 50 hpf. Representative pictures were captured from Video S1 and Video S2, respectively. Arrowheads indicate the budding cells. DA, dorsal aorta; AV, axial vein. (F) Statistical analyses of the frequency of HSPC generation. Data shown are the mean ± SEM, n = 3, *P < 0.01 by student's *t*-test. (G–O) The proliferation of HSPC is reduced in SUMOs or *Ubc9* morphants. Double immunostaining of *cmyb*-EGFP (G–I) and BrdU (J–L) in the CHT of Tg (*cmyb*-EGFP) line at 72 hpf. The bottom panel shows merged images (M–O). (P) Quantification of BrdU and *cmyb*-EGFP double positive cells in the CHT of Tg (*cmyb*-EGFP) line at 72 hpf. Data shown are the mean ± SD, n ≥ 3, *P < 0.01 by student's *t*-test.

HSPCs arise directly from the aortic endothelium during embryogenesis^15^,^16^,^17^. To assess whether the depletion of HSPCs in SUMO-deficient embryos was caused by improper vascular morphogenesis, the expression of *flk1*, a marker of endothelial cells, was examined. No overt differences were observed between control siblings and SUMOs or *Ubc9* morphants (Fig. 3 A–C), suggesting that the vascular system remains intact. Accordingly, a similar phenotype was also detected in the Tg (*flk1*-EGFP) transgenic line (data not shown). Next, we investigated whether the HSPC budding process was affected in SUMO-deficient embryos by confocal time-lapse imaging experiments. The results showed that the frequency of HSPC generation was profoundly decreased in SUMO-deficient embryos (0.1 cells per somite per hour, n = 3) compared to that of the control siblings (0.22 cells per somite per hour, n = 3) (Fig. 3 D–F; Supplementary video S1 and S2). To further explore whether cell apoptosis or proliferation was affected following HSPC generation, TUNEL and BrdU incorporation assays were performed in the Tg (*cmyb*-EGFP) transgenic line. Double immunostaining revealed no significant differences in apoptotic HSPC numbers between control siblings and SUMO-deficient embryos (Supplementary Fig. 2). In contrast, the percentage of *cmyb*-EGFP/BrdU double positive cells very significantly decreased in the CHT region of both SUMOs and *Ubc9* morphants compared with control siblings (Fig. 3 G–P). Taken together, these data suggest that the impairment of definitive hematopoiesis is attributed, at least in part, to reduced HSPC generation and subsequent hypoproliferation.

### Sumoylation of C/ebpα is implicated in HSPC development

C/ebpα is a member of the basic leucine zipper protein family of transcription factors, which contain a basic region (BR) and a leucine zipper domain at the C-terminus^18^,^19^. In the mouse haematopoietic system, C/ebpα not only plays a pivotal role in granulopoiesis^20^, but also regulates the self-renewal and proliferation of HSPC at a much earlier stage^21^,^22^,^23^,^24^. Both C/ebpα-deficient fetal and adult HSPCs exhibit enhanced proliferation and competitive repopulation activities^21^,^22^,^24^, whilst activation of C/ebpα in HSPCs leads to their reduced self-renewal and proliferation^23^. We have previously shown that zebrafish C/ebpα can be sumoylated, triggering transcriptional repression^9^. Thus, herein we investigated the potential role of C/ebpα sumoylation in the regulation of HSPC homeostasis.

**Figure 4:**
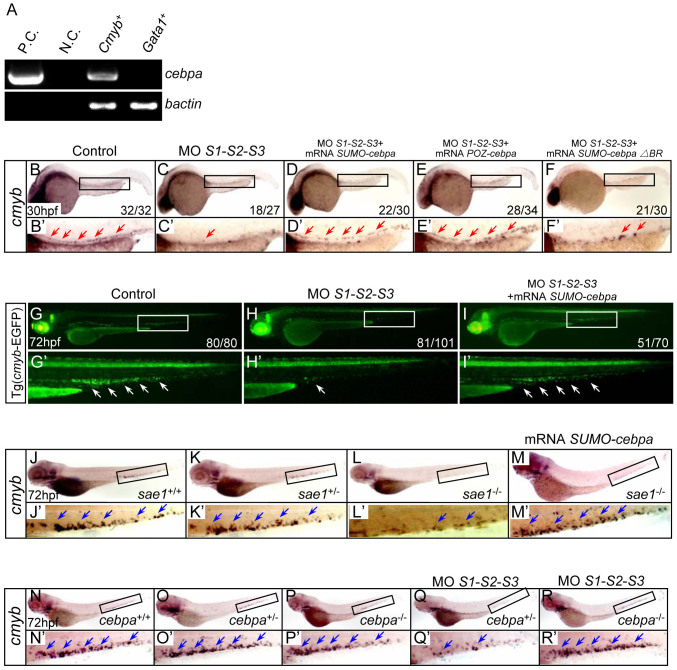
Sumoylation of C/ebpα is involved in HSPC development. (A) The expression of *cebpa* was examined by RT-PCR analysis. EGFP positive cells were sorted from the Tg (*cmyb*-EGFP) and Tg (*gata1*-EGFP) transgenic lines at 72 hpf, respectively, and subjected to RT-PCR. As a control, transcripts of the *bactin* gene were amplified. P.C., positive control (plasmid). N.C., negative control (H_2_O). (B–F) WISH assay of *cmyb* at 30 hpf. Boxed regions indicate the AGM. (B′–F′) Magnified images of corresponding boxed regions from B to F, respectively. Red arrows identify *cmyb*-positive cells in the AGM. (G–I) Fluorescent images of the Tg (*cmyb*-EGFP) line at 72 hpf. (G′–I′) Magnified images of corresponding boxed regions from G to I, respectively. White arrows identify *cmyb*-positive cells in the CHT. (J–M) WISH assay of *cmyb* in the *sae1* mutant embryos and siblings at 72 hpf. (N–R) WISH assay of *cmyb* in the *cebpa* mutant embryos and siblings at 72 hpf. (J′–R′) Magnified images of corresponding boxed regions from J to R, respectively. Blue arrows identify *cmyb*-positive cells in the CHT.

Firstly, to examine whether *cebpa* is expressed in HSPCs, homogenous EGFP positive cells were sorted from the Tg (*cmyb*-EGFP) transgenic line. In parallel, EGFP positive cells sorted from the Tg (*gata1*-EGFP) transgenic line served as a control. RT-PCR analysis showed that *cebpa* was expressed in the *cmyb*-EGFP positive cells, whilst no signal was detected in the *gata1*-EGFP positive cells (Fig. 4A), suggesting that *cebpa* may be expressed in the HSPCs of zebrafish, consistent with previous findings observed in mice^25^.

Secondly, to determine whether sumoylation of C/ebpα was involved in the defects of HSPC mediated by SUMO-deficiency, a series of rescue assays were carried out. Our previous work showed that, a SUMO-C/ebpα fusion protein, mimicking the constitutively sumoylated form of C/ebpα, and a POZ-C/ebpα fusion protein, mimicking the SUMO-mediated repressive form of C/ebpα, could significantly inhibit the transcriptional activity of wide type (WT) C/ebpα^9^. This allowed us to assess the rescue effects of the fusion proteins in the SUMO-deficient embryos. Critically, *in vivo* rescue assays showed that SUMO-C/ebpα or POZ-C/ebpα could effectively restore the SUMO-deficiency-mediated phenotypes (Fig. 4 B–E and Supplementary Fig. 3A), whereas SUMO-C/ebpα ΔBR (which lacks the basic region of C/ebpα and has no transcriptional activity and no repressive effect on the WT C/ebpα) (Supplementary Fig. 3B), was ineffective (Fig. 4F). Consistently, similar rescue effects using the fusion proteins were also observed in the Tg (*cmyb*-EGFP) transgenic line (Fig. 4 G–I). Overexpression of SUMO-C/ebpα, POZ-C/ebpα or SUMO-C/ebpα ΔBR alone had no obvious effect on HSPC development (Supplementary Fig. 3C). Collectively, these data suggest that sumoylation of C/ebpα is implicated in the phenotypes triggered by SUMO-deficiency.

Similarly, in the *sae1* mutation zebrafish line (encoding a key subunit of the E1 sumoylation pathway enzyme), systemic sumoylation was reduced and led to depletion of HSPCs in the CHT^12^. Again, SUMO-C/ebpα was also able to rescue the defects of HSPC in this mutant (Fig. 4 J–M), further confirming that sumoylation of C/ebpα is essential for the maintenance of HSPCs during definitive hematopoiesis.

To further confirm that sumoylation of C/ebpα is required for HSPC development, a *cebpa* mutant zebrafish line was generated using TALEN technology (Supplementary Fig. 4). There was no significant difference in the expression of *cmyb* between *cebpa* mutants and control siblings (Fig. 4 N–P). However, the expression of *cmyb* was only markedly reduced in SUMO-deficient *cebpa* heterozygous embryos (Fig. 4Q), and not changed in SUMO-deficient *cebpa* null homozygous embryos (Fig. 4R). These data demonstrate that C/ebpα is the major effector responsible for the defects triggered by SUMO-deficiency during definitive hematopoiesis in zebrafish.

### Runx1 is involved in C/ebpα sumoylation-dependent HSPC development

It has been reported that C/ebpα inhibits cell proliferation through regulating p21 protein stability^26^,^27^. To examine whether distinct C/ebpα sumoylation patterns could affect p21 protein stability, a p21 pulse-chase experiment was performed in the presence of either WT C/ebpα, unsumoylatable C/ebpα K125R or SUMO-C/ebpα. No significant differences in the p21 protein half-lives were observed (data not shown), suggesting that the defects of HSPC in SUMO-deficient embryos may not be due to the stabilization of p21.

**Figure 5:**
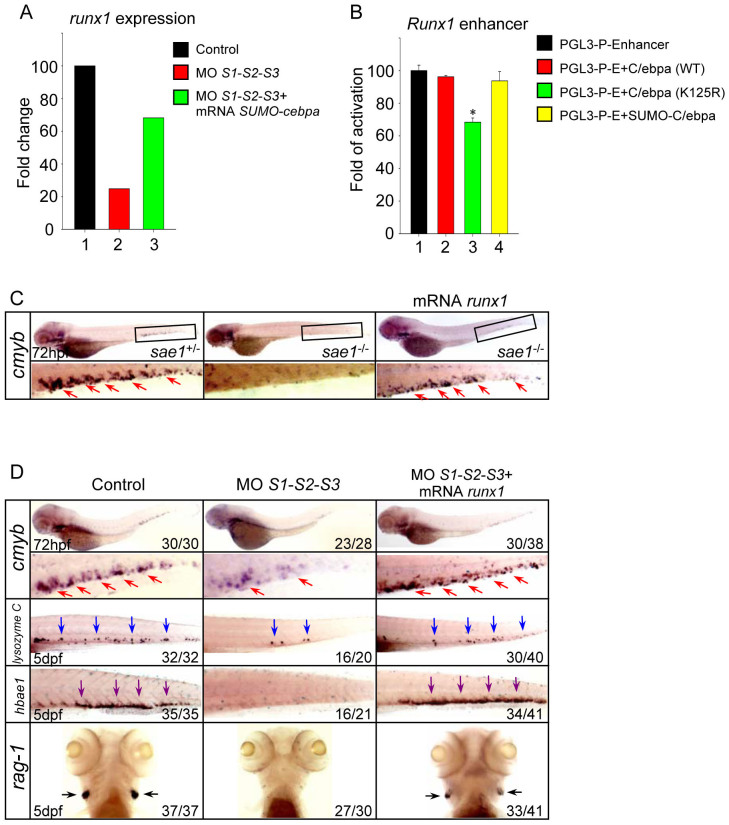
Runx1 is implicated in the hematopoietic defects triggered by SUMO-deficiency. (A) The relative expression level of *runx1* revealed by microarray analysis. The result shown is expressed as fold difference compared with the level (set to 100) detected in control embryos. (B) Luciferase activity assays were performed in 293T cells using the various C/ebpα constructs indicated. The Renilla plasmid was used as an internal control. Data shown are the mean ± SD of three independent experiments. *P < 0.05 by student's *t*-test. (C) WISH assay of *cmyb* in the *sae1* mutant embryos and siblings at 72 hpf. (D) WISH assay of *cmyb* at 72 hpf and *lysozyme C*, *hbae1* and *rag1* at 5 dpf. Note that *runx1* overexpression rescued the hematopoietic defects in the SUMO-deficient embryos. Red, blue, purple and black arrows identify *cmyb*, *lysozyme C*, *hbae1* and *rag1*-positive cells, respectively.

The basic region of C/ebpα plays a key role in the binding of DNA, which is essential for transcriptional activation (Supplementary Fig. 3A). The SUMO-C/ebpα ΔBR fusion protein, which lacks the basic region, was unable to rescue SUMO-deficient embryos, indicating that the transcriptional activity of C/ebpα is essential. To determine the potential downstream genes of C/ebpα, Affymetrix-based global gene expression analysis was performed on *cmyb*-positive HSPCs sorted from the Tg (*cmyb*-EGFP) line injected with either SUMO MOs or SUMO MOs plus *SUMO-cebpa* mRNA. Microarray analysis showed that the expression of *runx1*, a key regulator of definitive hematopoiesis^28^, was down-regulated in SUMOs morphants, while SUMO-C/ebpα could restore its expression (Fig. 5A), implying that *runx1* might be regulated by the distinct sumoylation status of C/ebpα. In order to assess whether the sumoylation status of C/ebpα had a distinct effect on *runx1* expression, the mouse *Runx1* enhancer was cloned into a pGL3-Promoter vector^29^ and luciferase assays performed. The data revealed that C/ebpα K125R, the unsumoylatable form, significantly inhibited *Runx1* enhancer activity (Fig. 5B), demonstrating that desumoylated C/ebpα could negatively regulate *runx1* expression. To further investigate whether Runx1 was implicated in the defects of HSPC mediated by SUMO-deficiency *in vivo*, *runx1* mRNA was injected at the 1-cell stage in the *sae1* mutant embryos or SUMOs morphants. The results showed that Runx1 could efficiently rescue the reduced HSPCs both in the *sae1* mutants and SUMOs morphants (Fig. 5 C–D). Furthermore, the expression of lineage-specific markers was also restored (Fig. 5D). Thus, our data suggests that Runx1, negatively regulated by desumoylated C/ebpα, is implicated in the phenotypes triggered by SUMO-deficiency.

## Discussion

The biological function of sumoylation in hematopoiesis during early embryonic development has not been extensively studied. *Ubc9*-deficient mice die at the early postimplantation stage before hematopoiesis occurs^30^, precluding exploration of the functional role of sumoylation in hematopoiesis. Recently, a zebrafish mutant with defects in definitive hematopoiesis has been characterized. This phenotype is caused by a nonsense mutation of *sae1* gene, a subunit of the sumoylation pathway E1 enzyme^12^. However, the molecular mechanism involved is still poorly understood. In this study, we show that depletion of SUMO or Ubc9 leads to a decreased number of HSPCs during embryogenesis. The defects of definitive hematopoiesis are attributed primarily to reduced HSPC generation and proliferation. C/ebpα, as a key sumoylated target, is essential for HSPC development during definitive hematopoiesis in zebrafish. These defective HSPCs triggered by hyposumoylation are probably caused by a cell-autonomous effect, as demonstrated in published chimera generation experiments^12^.

The transcription factor C/ebpα is a critical factor for granulopoiesis^20^. C/ebpα regulates the expression of a number of myeloid-specific genes^31^,^32^,^33^,^34^. Our previous work showed that C/ebpα desumoylation increased its transcriptional activity and promoted myelopoiesis of myelo-erythroid progenitor cells during primitive hematopoiesis^9^. Recently, an increasing number of reports have implicated C/ebpα as an important modulator of HSPCs function^21^,^22^,^23^,^24^. In mice, C/ebpα-deficient HSPCs possess enhanced proliferation and repopulation activity^21^,^22^,^24^, while C/ebpα activation in HSPCs results in their reduced self-renewal and proliferation^23^, suggesting a role for C/ebpα in limiting HSPC proliferation. Here we demonstrate for the first time that, under physiological conditions, desumoylation-activated C/ebpα leads to reduced proliferation of HSPC during zebrafish definitive hematopoiesis, thus highlighting the conserved role of C/ebpα in HSPC regulation across vertebrate species.

**Figure 6:**
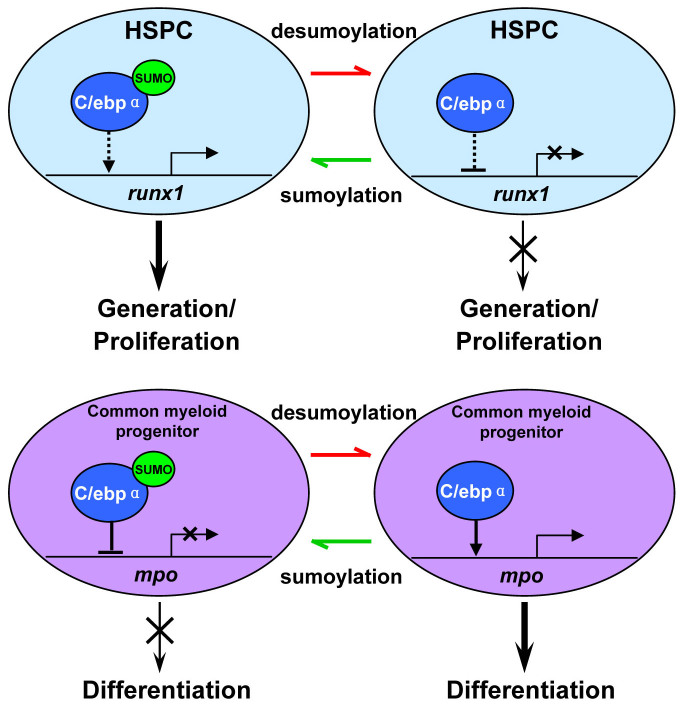
Schematic depiction of the distinct functions of C/ebpα sumoylation in the regulation of HSPC and myeloid progenitor development during hematopoiesis. HSPC homeostasis is maintained by the fine-tuning of C/ebpα sumoylation. Sumoylated C/ebpα de-represses *runx1* transcription, which in turn participates in regulating normal HSPC development (top panel, left). In SUMO-deficient embryos, C/ebpα is desumoylated, which subsequently inhibits Runx1 activity, likely through direct transcriptional repression. As a consequence, HSPC generation and proliferation is reduced (top panel, right). In contrast, during myeloid cell development, inactivation of C/ebpα triggered by sumoylation blocks myeloid progenitor differentiation (bottom panel, left), while the activated form triggered by desumoylation promotes myelopoiesis (bottom panel, right).

Mechanistically, our microarray analysis identified that upon the loss of SUMOs, *runx1* (a critical transcription factor capable of regulating HSPC induction and proliferation^35^) was strongly down-regulated. Yet, SUMO-C/ebpα could largely restore its expression and rescue the definitive HSPC defects. Furthermore, *runx1* mRNA was also able to rescue the hematopoietic defects in the SUMO-deficient embryos, implying that *runx1* might be a downstream gene of C/ebpα. Based on our studies and those of others, we hypothesized that C/ebpα may exert distinct functions depending on its sumoylation status and specific given cell compartment (Fig. 6). The role of C/ebpα sumoylation in HSPCs appears to be quite distinct from its function in myeloid progenitor cells development. Within HSPCs, desumoylated C/ebpα acts as a transcriptional repressor and down-regulates *runx1* directly or indirectly, which results in the attenuated HSPC generation and proliferation. Under physiological conditions, sumoylated C/ebpα, through an as yet unknown mechanism, likely promotes protein structural changes, de-represses *runx1* transcription, and ultimately participates in controlling normal HSPC development (Fig. 6, top panel). In contrast, during myeloid development, the desumoylation-activated C/ebpα is required to promote myeloid progenitor cells differentiation through activation of myeloid-specific gene expression (Fig. 6, bottom panel).

ChIP assays aimed to verify whether *runx1* expression was directly regulated by C/ebpα. Unfortunately however, due to both a lack of an endogenous zebrafish C/ebpα specific antibody, and *cebpa* mRNA overexpression causing embryonic death during gastrulation prior to the onset of hematopoiesis, further studies were not feasible. Nevertheless, it has been demonstrated by other groups that C/ebpα can bind to the *runx1* promoter in both U937 cells and K562 cells^36^. Further work to investigate the ability of C/ebpα to regulate *runx1* expression in zebrafish HSPCs is envisioned.

In summary, we provide novel evidence that sumoylation of C/ebpα is essential for HSPC development and demonstrate that protein posttranslational modification participates in the maintenance of HSPC properties during definitive hematopoiesis in zebrafish. Our studies shed new light on the role of protein sumoylation in HSPC regulation and may provide rationale for targeting of SUMO pathway in hematologic disorders.

## Methods

### Zebrafish

Zebrafish maintenance and staging were performed as described previously^37^. The transgenic lines, Tg (*cmyb*:EGFP)^38^, Tg (*mpo*:EGFP)^9^, Tg (*gata1*:EGFP)^39^ and Tg (*flk1*:EGFP), and *sae1* mutants^12^, p53 mutants^40^ were used. The zebrafish facility and study were approved by the Institutional Review Board of the Institute of Health Sciences, Shanghai Institutes of Biological Sciences, Chinese Academy of Sciences (Shanghai, China) and the methods were carried out in accordance with the approved guidelines.

### Generation of constructs

The mouse *Runx1* enhancer^29^ and zebrafish *runx1* were amplified by RT-PCR with indicated primers (Supplementary table S1), then cloned into pGL3-Promoter vector and pCS2+ vector, respectively. *C/ebpα ΔBR* and *SUMO-C/ebpα ΔBR* was generated with indicated primers, respectively (Supplementary table S1).

### Morpholinos and mRNAs microinjection

MOs were purchased from Gene Tools. SUMO1 MO GTCTCCGTGTCTGACATGATATTCC; SUMO2 MO CATGGTTATTGTATTTGCGCTTCTC; SUMO3 MO TAGGCTTGTCTTCGGACATTTTTGC; *Ubc9.1* MO TCAGAGCAATGCCAGACATGACCAC; *Ubc9.2* MO GACGACTCAATGCTATACCAGACAT; *p53* MO TCTTGGCTGTCGTTTTGCGCCATTG. The doses of injection per embryo were: SUMO1-SUMO2-SUMO3 MOs combination, 4.15 ng each; *Ubc9.1-Ubc9.2* MOs combination, 4.15 ng each; *p53* MO, 4.15 ng. Capped mRNAs were transcribed from linearized pCS2+ plasmid (mMessage Machine, Ambion), purified and diluted to 50 ng/μl (*SUMO-cebpα*, *SUMO-cebpα ΔBR*, *POZ-cebpα* and *runx1* mRNA) for microinjection.

### Whole-mount mRNA *in situ* hybridization

Digoxigenin-labeled antisense RNA probes were transcribed from linearized constructs using T3 or T7 polymerase (Roche). Whole-mount mRNA *in situ* hybridization was performed as described previously^9^. The probes were detected using alkaline phosphatase (AP)-coupled anti-digoxigenin Fab fragment antibody (Roche) with BCIP/NBT staining (Vector Laboratories).

### Time-lapse confocal fluorescence imaging

Olympus FV 1000 confocal microscopy was used for the four-dimensional time-lapse fluorescence imaging^15^,^16^. Z stacks were taken every 2 min from 30 to 50 hpf. Videos were created after processing with the FV10-ASW version3 software.

### Bromodeoxyuridine (BrdU) labeling assay, TUNEL assay and double immunostaining

Briefly, Tg(*cmyb*:EGFP) embryos were incubated with 10 mM BrdU (Sigma) for 30 minutes and incubated with egg water for another 2 hours, then fixed in 4% paraformaldehyde (PFA) at 3 dpf. After dehydration and rehydration, the embryos were treated with Proteinase K (10 mg/ml, Finnzyme) for 30 minutes and re-fixed in 4% PFA for 30 minutes. After blocking with blocking buffer (2 mg/ml BSA, 10% FBS, 0.3% Triton-X100 and 1% DMSO in PBST), the embryos were stained with rabbit anti-GFP (Invitrogen) primary antibody and Alexa Fluor 488-conjugated anti-rabbit (Invitrogen) secondary antibodies. The embryos were fixed again, treated with Proteinase K for the second time, and re-fixed in 4% PFA. The embryos were then incubated with 2 N HCl for 1 hour and stained with mouse anti-BrdU (Roche) and rabbit anti-GFP (Invitrogen) antibodies. Finally, Alexa Fluor 594-conjugated anti-mouse and Alexa Fluor 488-conjugated anti-rabbit (Invitrogen) secondary antibodies were used. Images were taken using Olympus FV 1000 confocal microscopy equipped with the FV10-ASW version3 software.

Terminal transferase UTP nick end labeling (TUNEL) was performed using the In Situ Cell Death Detection Kit, TMR red (Roche) according to the manufacturer's recommendations.

### Luciferase reporter assay

293 T cells were transfected with indicated plasmids using Effectene Transfection Reagent (QIAGEN). Cells were harvested 36 hours after transfection and luciferase activities were analyzed using the Dual Luciferase Reporter Assay Kit (Promega) according to the manufacturer's protocols. Luciferase activity was normalized to Renilla activity.

### Generation of TALEN-mediated *cebpa* mutant zebrafish

TAL Effector Nucleotide Targeter software was used to design candidate TALEN target site for *cebpa* gene^41^. TALEN expression vectors were constructed as previously described^42^. The corresponding mRNAs were prepared using SP6 mMESSAGE mMACHINE Kit (Ambion) and were injected in pairs into one-cell stage zebrafish embryos at the dosage of 150 pg (for each TALEN, 300 pg in total). To examine the targeting effect of *cebpa* TALENs, genomic DNA of 10 normally developing embryos was extracted as a pool at 30 hpf. A 867 bp DNA fragment containing the TALEN target site was amplified by PCR (forward primer: 5′-ATGGAGCAAGCAAACCTCTACGAGG-3′, reverse primer: 5′-TTAAGCGCAGTTGCCCATGGCTTTG-3′). 10 μl of the PCR product was digested by *Sal* I at 37°C overnight and fractionated through 2.5% agarose gel. The uncleaved band was recovered after gel electrophoresis and cloned for sequencing analysis. To screen the heritable mutants, the injected F0 founder embryos were raised to adulthood and outcrossed with wild type zebrafish. From each cross, genomic DNA of 10 F1 embryos was extracted as a pool and analyzed via PCR amplification and sequencing as described above. Siblings of F1 embryos carrying potential indel mutations were raised to adulthood. Genomic DNA was isolated from tail clips of these F1 fish and the status of the target site was analyzed via PCR amplification and sequencing to identify mutants. F1 zebrafish carrying 2 bp deletion in target site and their offspring were used for further study.

### Sudan black staining

Sudan black staining was performed as described previously^43^.

### Cell sorting and microarray

About 400 *cmyb*-EGFP transgenic embryos at 72 hpf were collected and digested with 0.5% trypsin (GIBCO) for 15 minutes at 37°C. Single-cell suspension was obtained by centrifugation at 400 g for 5 minutes, washing twice with PBS, and passing through a 40 μm nylon mesh filter. Fluorescence-activated cell sorting was performed with MoFlo FACS (DakoCytomation) to obtain homogenous EGFP positive cells. Then, RNA was extracted using Trizol (Invitrogen), which were subsequently subject to RT-PCR or microarray (Shanghai Biochip Co. Ltd).

## Author Contributions

H.Y. designed the research, performed experiments, analyzed data and wrote the manuscript; T.Z., X.H.L. and M.D. performed experiments; W.Q.Z., Z.L.W., S.J.C., Z.C. and H.d.T. provided suggestions on experimental design and analyzed data; J.Z. and J.Z. designed experiments, analyzed data and wrote the manuscript.

## Supplementary Material

Supplementary Information Supplementary Files

Supplementary Information Supplementary Video S1

Supplementary Information Supplementary Video S2
