# Supplementary material for: Sumoylation of CCAAT/enhancer-binding protein α is implicated in hematopoietic stem/progenitor cell development through regulating runx1 in zebrafish
Source: Sci Rep. 2015 Mar 11;5:9011. doi: 10.1038/srep09011 (PMC4355724; doi:10.1038/srep09011)
Supplement: Supplementary Information — Supplementary Files [file srep09011-s1.pdf]

Sumoylation of CCAAT/enhancer-binding protein α is implicated in hematopoietic stem/progenitor cell development through regulating *runx1* in zebrafish

Hao Yuan[1], Tao Zhang[1], Xiaohui Liu[1], Min Deng[2], Wenqing Zhang[3], Zilong Wen[4], Saijuan Chen[1], Zhu Chen[1], Hugues de The[1, 5], Jun Zhou[1, *] and Jun Zhu[1, 5, *]

Supplementary Figure Legends

Figure S1. HSPCs are reduced in SUMO-deficient embryos. (A) WISH assay of *runx1* at 30 hpf. Boxed regions indicate the AGM. Blue arrows identify *runx1*-positive cells in the AGM. (B) Fluorescent images of the Tg (*cmyb*-EGFP) line at 72 hpf. Boxed regions indicate the CHT. White arrows identify *cmyb*-positive cells in the CHT. (C) Quantification of *cmyb*-EGFP positive cells in the CHT region of Tg (*cmyb*-EGFP) line at 72 hpf. Data shown are the mean ± SD, n≥3, **P*<0.01 by student's *t*-test. (D) WISH assay of *cmyb* in the *p53* mutant embryos at 72 hpf.

Figure S2. Apoptosis of HSPC is not affected in SUMO-deficient embryos. (A-I) Double immunostaining of *cmyb*-EGFP (A-C) and TUNEL (D-F) in the CHT of Tg (*cmyb*-EGFP) line at 72 hpf. The bottom panel shows merged images (G-I). (J) Quantification of TUNEL and *cmyb*-EGFP double positive cells in the CHT of Tg (*cmyb*-EGFP) line at 72 hpf. Data shown are the mean ± SD, n≥5, P values stand for student's *t*-test.

Figure S3. WISH and Luciferase activity assays. (A) WISH assay of *runx1* at 36 hpf.

Overexpression of *SUMO-cebpa* could rescue the HSPC defects of SUMOs morphant. Blue arrows identify *runx1*-positive cells in the AGM. (B) Luciferase activity assays were performed in 293T cells using various C/ebpα constructs indicated. The Renilla plasmid was used as an internal control. Note that SUMO-C/ebpα △BR lost the repressive effect on the transcriptional activity of WT C/ebpα. (C) WISH assay of *cmyb* at 72 hpf. Overexpression of *SUMO-cebpa*, *POZ-cebpa* or *SUMO-cebpa △BR* alone had no obvious effect on HSPC development.

Figure S4. Targeted disruption of *cebpa* gene with TALENs in zebrafish. (A) Partial structure and sequence of the *cebpa* gene, showing the target sites (underlined) of the left and right TALENs. The *Sal* I site used for detecting mutations is highlighted in red. (B) Sequencing results of *cebpa* mutation induced by TALENs in F1 fish line. The TALENs binding sites are underlined. Deletions are indicated by dashes. (C) Sudan black staining of embryos from incross of heterozygous *cebpa* mutants at 72 hpf. Red arrows indicated Sudan black positive cells in the CHT. (D) Genotyping of the corresponding embryos in (C) by genomic PCR amplification and *Sal* I digestion.

Figure S1.

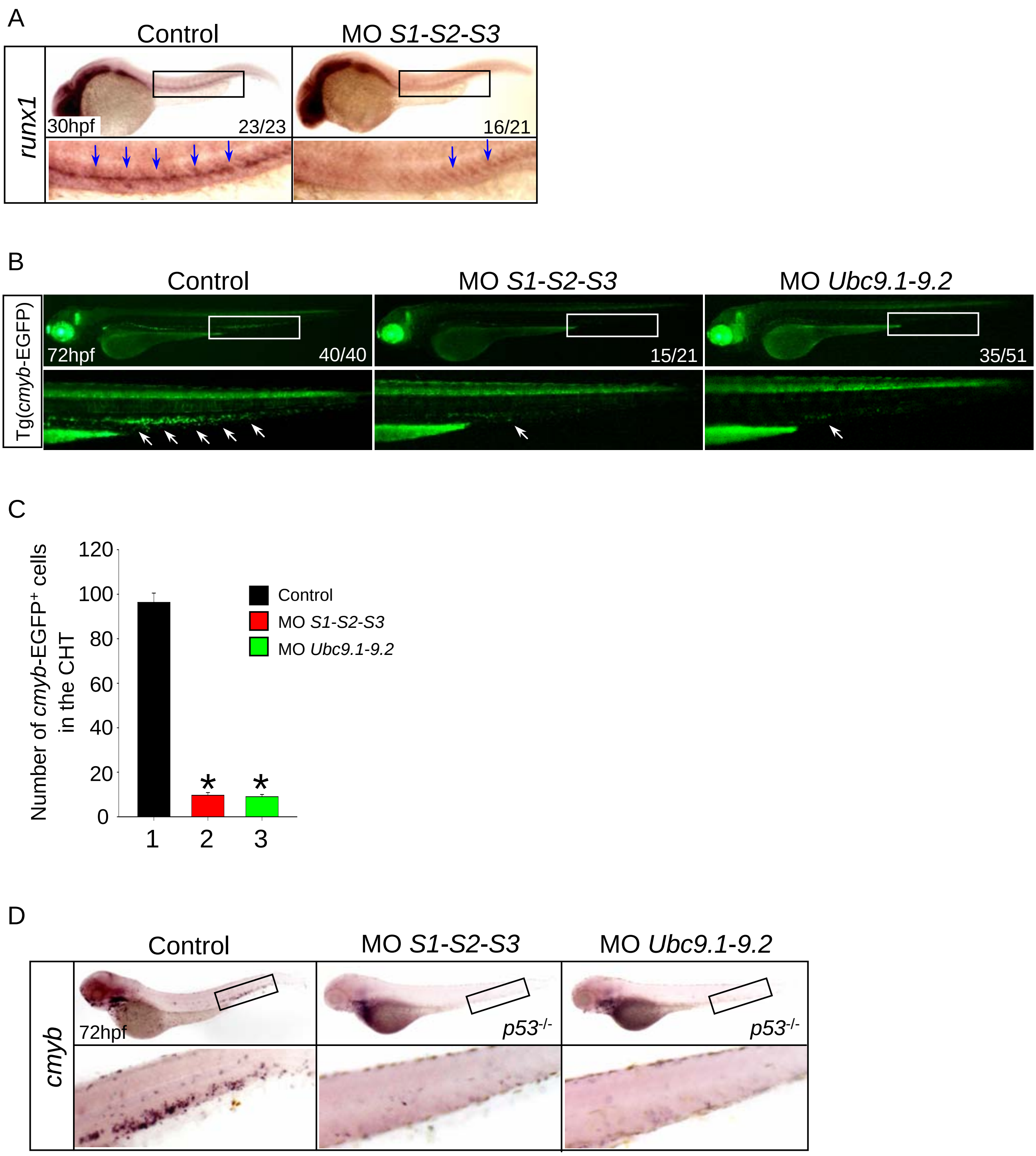
A
Control
MO S1-S2-S3
runx1
30hpf
23/23
16/21
B
Control
MO S1-S2-S3
MO Ubc9.1-9.2
Tg(cmyb-EGFP)
72hpf
40/40
15/21
35/51
C
Number of cmyb-EGFP+ cells in the CHT
120
100
80
60
40
20
0
1
2
3
Control
MO S1-S2-S3
MO Ubc9.1-9.2
D
Control
MO S1-S2-S3
MO Ubc9.1-9.2
cmyb
72hpf
p53-/-
p53-/-

Figure S2.

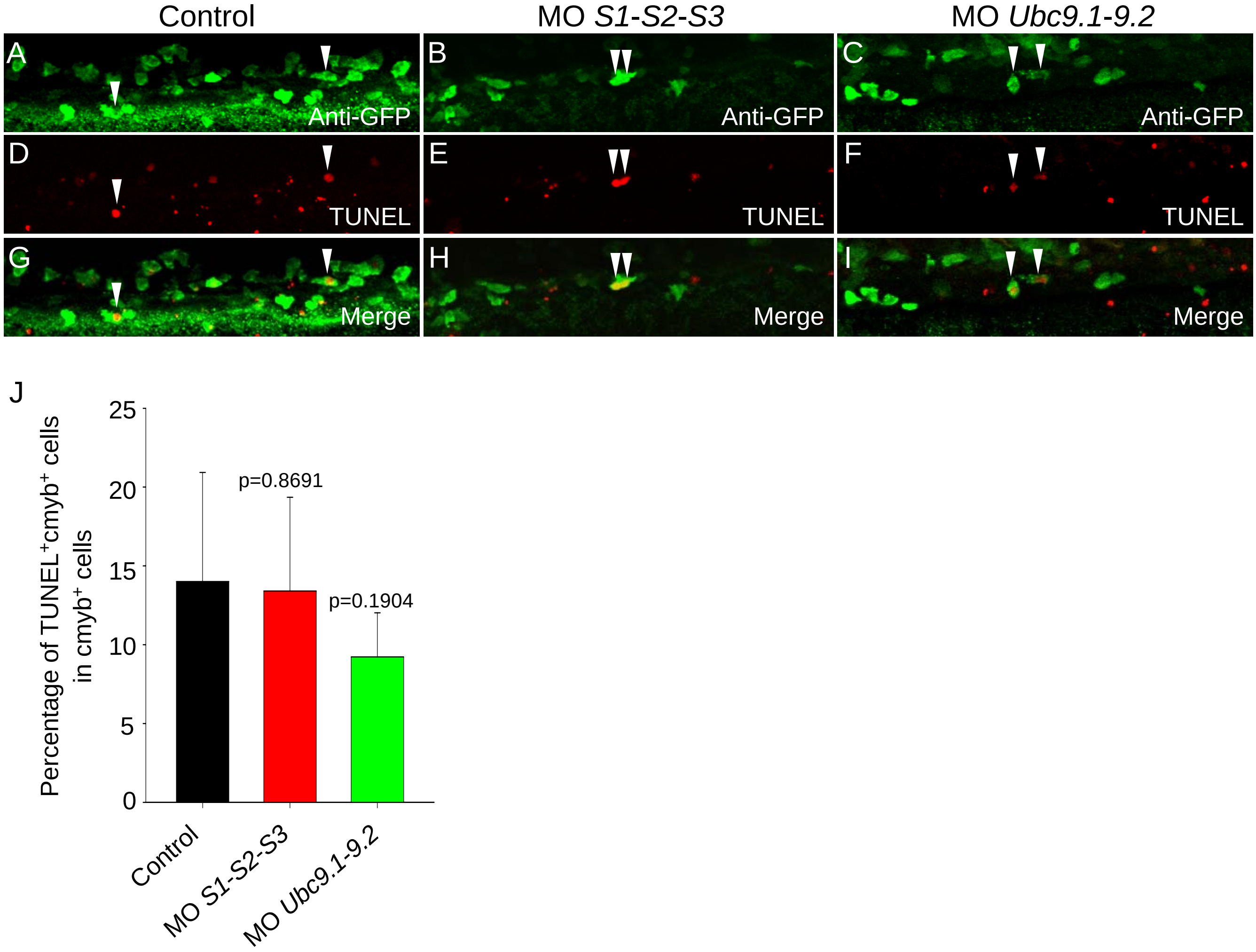

Control
MO *S1-S2-S3*
MO *Ubc9.1-9.2*
A
Anti-GFP
B
Anti-GFP
C
Anti-GFP
D
TUNEL
E
TUNEL
F
TUNEL
G
Merge
H
Merge
I
Merge
J
Percentage of TUNEL+cmyb+ cells in cmyb+ cells
25
20
15
10
5
0
p=0.8691
p=0.1904
Control
MO S1-S2-S3
MO Ubc9.1-9.2

Figure S3.

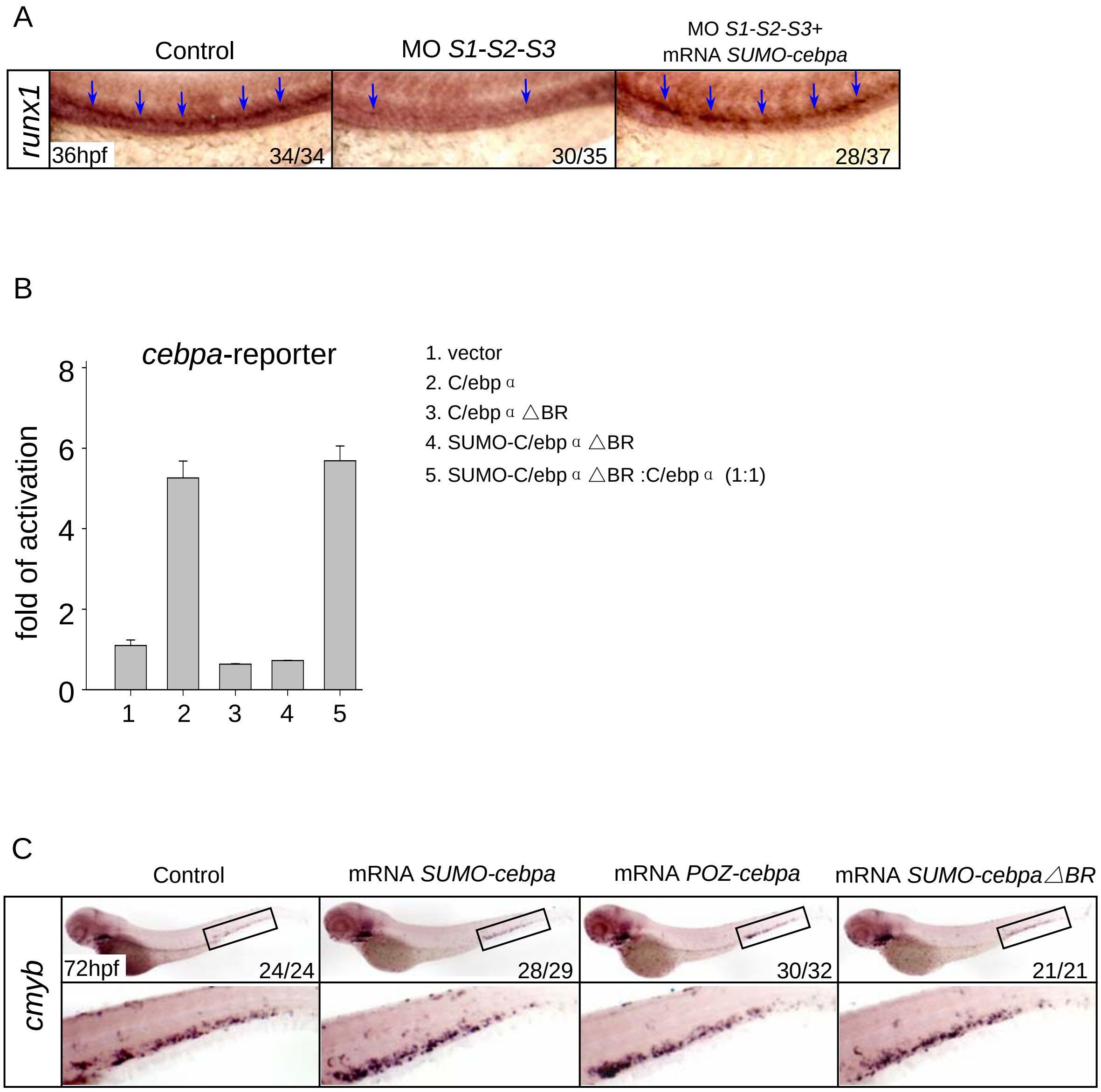
A
Control
MO S1-S2-S3
MO S1-S2-S3+
mRNA SUMO-cebpa
runx1
36hpf
34/34
30/35
28/37
B
cebpa-reporter
fold of activation
0
2
4
6
8
1
2
3
4
5
1. vector
2. C/ebpα
3. C/ebpα △BR
4. SUMO-C/ebpα △BR
5. SUMO-C/ebpα △BR :C/ebpα (1:1)
C
Control
mRNA SUMO-cebpa
mRNA POZ-cebpa
mRNA SUMO-cebpa△BR
cmyb
72hpf
24/24
28/29
30/32
21/21

Figure S4.

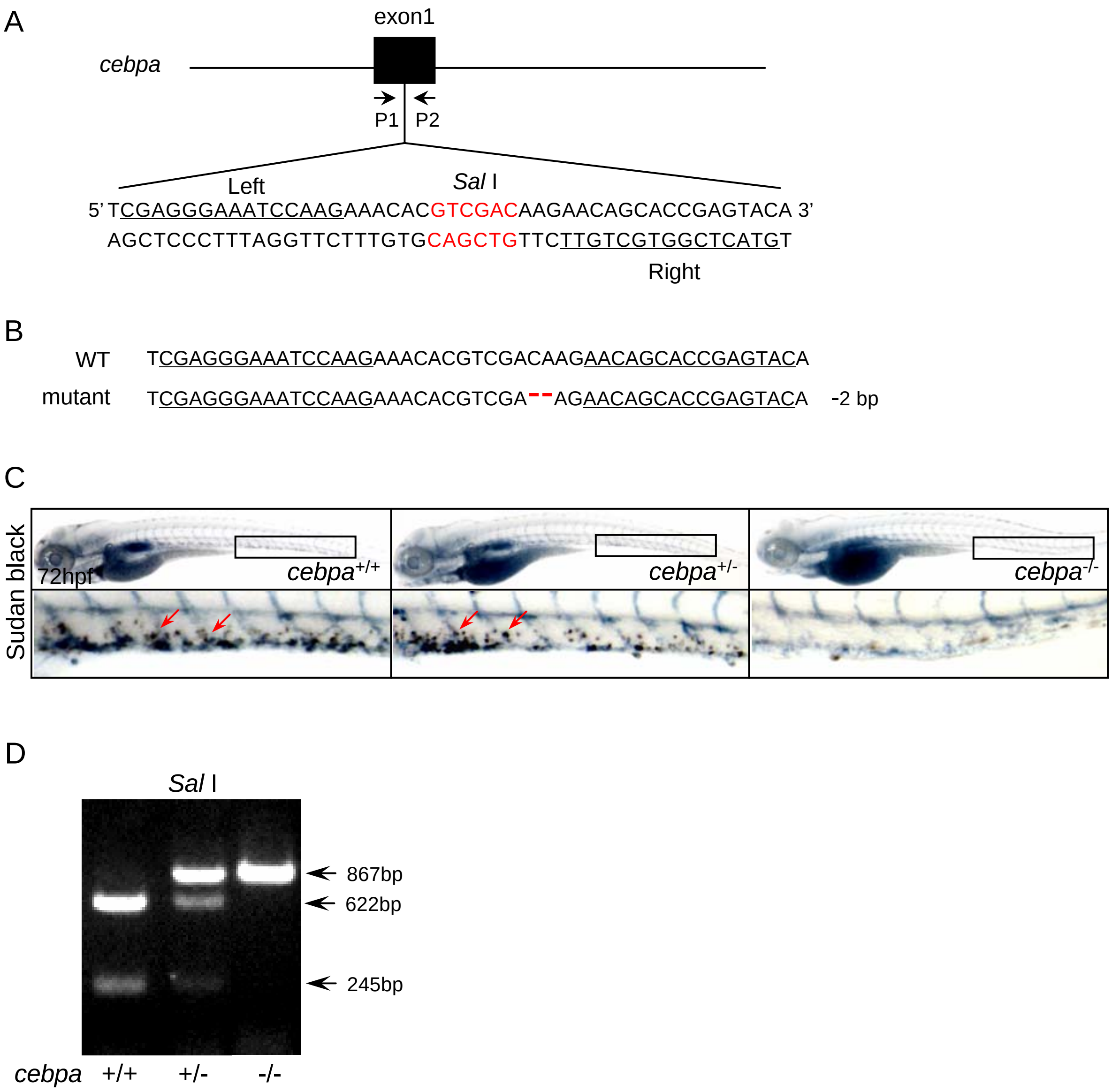
A
exon1
cebpa
P1
P2
Left
Sal I
5’ TCGAGGGAAATCCAAGAAACACGTCGACAAGAACAGCACCGAGTACA 3’
AGCTCCCTTTAGGTTCTTTGTGCAGCTGTTCTTGTCGTGGCTCATGT
Right
B
WT TCGAGGGAAATCCAAGAAACACGTCGACAAGAACAGCACCGAGTACA
mutant TCGAGGGAAATCCAAGAAACACGTCGA--AGAACAGCACCGAGTACA -2 bp
C
Sudan black
72hpf
cebpa+/+
cebpa+/-
cebpa-/-
D
Sal I
867bp
622bp
245bp
cebpa +/+ +/- -/-

Table S1. Primer sequences

Primer name		Primer sequence
mouse *Runx1* enhancer	Forward	GAGGATCCGGGGTGGGAGGTGTAAGTTC
	Reverse	GAGTCGACCAGGTGTCAGCAACCCAT
runx1	Forward	CCGGAATTCATGGACTACAAGGACGACGATGACAAAGTTTTTCTTTGGGACGCCAA
	Reverse	CCGCTCGAGGCTGTCAGTATGGCCTCCAG
C/ebpα △BR	Forward	GGAGCGCAACAACATAGCCGTGAATGTGGAGACGCAACAAAAAG
	Reverse	CTTTTTGTTGCGTCTCCACATTCACGGCTATGTTGTTGCGCTCC
SUMO2-C/ebpα △BR	Forward	GGAGCGCAACAACATAGCCGTGAATGTGGAGACGCAACAAAAAG
	Reverse	CTTTTTGTTGCGTCTCCACATTCACGGCTATGTTGTTGCGCTCC
cebpa	Forward	ATGGAGCAAGCAAACCTCTACGAGG
	Reverse	TTAAGCGCAGTTGCCCATGGCTTTG
bactin	Forward	GATCTTCACTCCCCTTGTTCA
	Reverse	GGCAGCGATTTCCTCATC
